# Newborn screening for sickle cell disease in Butembo and Beni: a pilot experience in a highland region of the Democratic Republic of Congo

**DOI:** 10.11604/pamj.2023.45.56.36087

**Published:** 2023-05-24

**Authors:** Mupenzi Mumbere, Salomon Batina-Agasa, Naura Apio Uvoya, Emmanuel Tebandite Kasai, Paul Kambale Kombi, Roland Marini Djang'eing'a, Jean-Pierre Alworong'a Opara

**Affiliations:** 1 Department of Pediatrics, Faculty of Medicine, Catholic University of Graben, Butembo, Democratic Republic of the Congo,; 2 Department of Internal Medicine, Faculty of Medicine and Pharmacy, University of Kisangani, Kisangani, Democratic Republic of the Congo,; 3 Department of Pediatrics, Faculty of Medicine, University of Bunia, Bunia, Democratic Republic of the Congo,; 4 Department of Pediatrics, Faculty of Medicine and Pharmacy, University of Kisangani, Kisangani, Democratic Republic of the Congo,; 5 Department of Pharmaceutical Sciences, Laboratory of Analytical Chemistry, Faculty of Medicine, University of Liège, Liège, Belgium

**Keywords:** Newborn screening, sickle cell disease, Beni, Butembo, Democratic Republic of the Congo

## Abstract

**Introduction:**

sickle cell disease is an inherited autosomal recessive hemoglobin disorder resulting in acute and chronic systemic complications. Despite the high burden of sickle cell disease in the Democratic Republic of the Congo, limited data on disease prevalence is available and systematic screening is not offered to newborns. This study aimed to provide neonatal prevalence and associated factors to the phenotypic manifestation of sickle cell disease in an eastern region of the Democratic Republic of the Congo.

**Methods:**

the study was conducted from 20^th^ April 2021 to 20^th^ January 2022 in the cities of Beni and Butembo, involving live full-term newborns whose parents consented to participate. Blood was taken with heel pricks and analyzed using the point-of-care diagnostic tool HemoTypeSC™. We used Fisher´s exact test to compare frequencies between groups. P-value <0.05 was considered statistically significant. **Results:** of the 1195 newborns screened, 1122 (93.9%) were tested as having hemoglobin AA, 71 (5.9%) hemoglobin AS, 2 (0.2%) hemoglobin SS and none hemoglobin C. The mother´s ethnicity was significantly associated with the phenotypic expression of sickle cell disease.

**Conclusion:**

sickle cell disease prevalence is lower in Butembo and Beni than in other regions of the Democratic Republic of the Congo. However, it remains an alarming public health issue. Systematic newborn screening, parent/patient education and early management programs constitute an urgent need to be addressed by decision-makers.

## Introduction

Sickle cell disease (SCD) is an inherited autosomal recessive hemoglobin (Hb) disorder caused by the replacement of normal Hb (HbA) by mutant Hb (sickle Hb, HbC, etc.) resulting in acute and chronic systemic complications [1]. On deoxygenation, sickle haemoglobin undergoes a conformational change that promotes intracellular polymerisation, which leads to distortion of the normal biconcave erythrocyte disc into the distinctive and pathological crescent shape. This abnormal shape results in haemolytic anaemia, recurrent vaso-occlusion and organ damage that together cause substantial morbidity and early mortality [2]. SCD is a globally distributed genetic blood disorder of high prevalence in regions where malaria is endemic [3]. Each year in Africa, approximately 300 000 individuals are born with SCD [4]. A study carried out in five countries (Burkina Faso, Democratic Republic of the Congo, Côte d´Ivoire, Mali, and Senegal) showed mortality estimates attributable to SCD of 15.3% for children younger than 1 year, 36.4% for those younger than 5 years, and 43.3% for those younger than 10 years [5].

The Democratic Republic of the Congo (DRC), the second-largest African country, is also the second African country most affected by SCD after Nigeria [6]. There is no recent large-scale newborn screening (NBS) data. However, based on some previous studies, estimates vary from 5 to 40% and 0.96 to about 2% for sickle cell trait (HbAS) and homozygous SCD (HbSS), respectively, with notable geographic variations [7-9]. In the northeastern region of the DRC, to the best of our knowledge, to date, newborn screening for SCD has never been carried out. While in developed countries, NBS and early comprehensive patient care have been proven to be effective in reducing disease severity and mortality [10]; unfortunately, none of these measures are systematically implemented in the DRC. The present study aimed to conduct a NBS of SCD in the cities of Butembo and Beni using a validated point-of-care (POC) diagnostic tool, HemoTypeSC™. The study was motivated by the lack of data on sickle cell disease prevalence in an area whose climatic characteristics, marked by intense rainfall, lower temperatures and higher altitude, do not favour malaria proliferation, which is traditionally associated with a higher prevalence of sickle cell disease in the world, particularly in Africa.

## Methods

**Figure 1:**
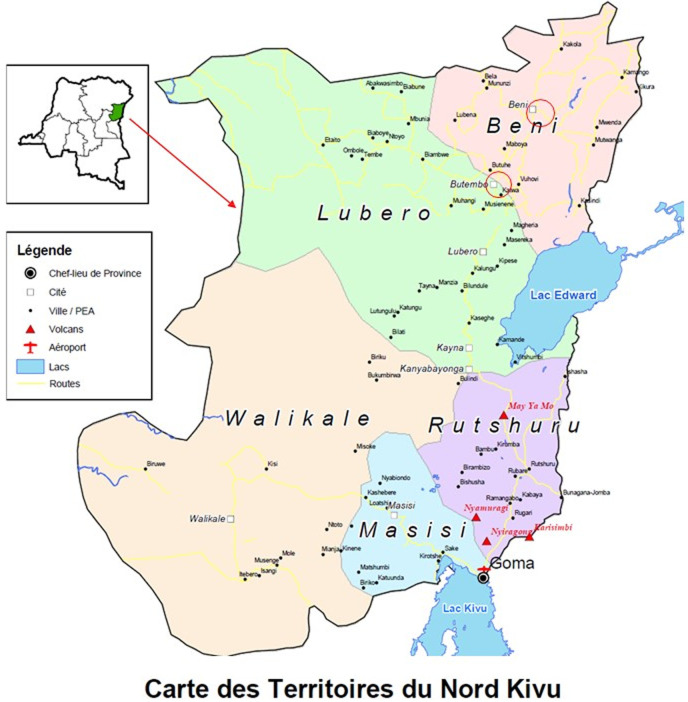
geographical location of the cities of Beni and Butembo in the province of North-Kivu, Northeastern DRC

**Study design and setting:** this is a descriptive cross-sectional study that was conducted in the cities of Butembo and Beni, located in the province of North-Kivu and distant of 50 km (Figure 1). In 2021, these cities counted 762 523 and 375 289 inhabitants, respectively (Annual report of the Health Districts of Beni, Butembo and Katwa). Data were collected in maternity wards of the following major hospitals during 9 months (20^th^ April 2021- 20^th^ January 2022): Beni General Referral Hospital, Katwa General Referral Hospital, Matanda Hospital and University Clinics of Graben.

**Inclusion and exclusion criteria:** we included healthy, transfusion-naïve neonates aged 0-28 days with gestational terms between ≥ 37 and < 42 weeks in the study. We excluded those with incomplete data from the analysis.

### Data collection

Prior to data collection, the principal investigator trained a team of four nurses in data collection according to standard operational procedures. On a weekly basis, he performed overall evaluation to ensure that data collection was done in accordance with the procedures and whether the data were complete, consistent and accurate according to data integrity rules. The study team offered pre-screening information on SCD to mothers in local languages, namely Kinande and/or Kiswahili as appropriate, during antenatal care visits and in maternity wards after delivery, in order to obtain their informed consent before data collection. We tested blood samples with HemoTypeSC™. The HemotypeSC is a competitive side-flow immunoassay that uses monoclonal antibodies to detect from whole blood, the hemoglobin phenotypes Hb AA (normal), Hb SS (homozygous sickle cell disease S) and Hb CC (homozygous sickle cell disease C), Hb SC (composite heterozygous sickle cell disease), Hb AS and Hb AC (sickle cell trait or heterozygous sickle cell disease). The interpretation of the test is different from many other rapid tests in that for HemoTypeSC, the presence of a line indicates the absence of the corresponding hemoglobin type. It is the absence of a line that indicates the presence of the hemoglobin type. In various field settings, it showed very good sensitivity and specificity profiles compared to standard diagnostic tools (>99%) [11-14].

Capillary blood (a tiny drop of blood equivalent to 1.5 µL) was collected with heel pricks and immediately tested using the HemoTypeSC™ rapid diagnostic test (Silver Lake Research Corporation, Azusa, CA, USA). We abided to the manufacturer´s instructions [15]: 1) Using a dropper pipette, add six drops of distilled water to the test vial. Place the test vial in a compatible rack. 2) Open vial of blood sampling devices, remove one blood sampling device and reclose vial. Obtain blood sample, touch the white pad of the blood sampling device to the blood sample, until the white pad absorbs the blood droplet. Ensure that the entire white pad has turned red. 3) Insert blood sampling device into the test vial water and swirl to mix (check visually to ensure that water has become pink or light-red in color). 4) Open vial of test strips, remove one test strip and reclose vial. Insert HemoTypeSC™ test strip into the test vial with arrows pointing down. 5) Wait 10 minutes. 6) Take HemoTypeSC™ test strip out of the test vial and read results. Compare test strip to the manufacturer´s results chart for reference. Results were immediately communicated to the parents in accordance with medical confidentiality. In order to attain our study objectives, we systematically collected the following variables for each screened participant: 1) neonates´ information: gender, age, birth weight, length and head circumference, presence/absence of visible congenital anomalies; 2) mothers´ information: age, marital status (legally/religiously married or not), ethnicity, number of SCD patients in siblings, history of SCD testing.

**Study sample:** for convenience, we selected the four main hospital facilities in our study area to screen all the newborns who met the inclusion criteria during the period defined for our study. In total, 1195 newborns were screened.

**Data analysis:** we used Epi Info version 7.2.4.0 for data entry and the RStudio version 3.6.3 for data analysis. We used proportions for categorical variables and means for normal variables. To compare the frequencies of studied variables between Hb phenotypes groups, we used the Fisher´s exact test. A P-value < 0.05 was considered to indicate statistical significance.

### Ethical considerations

Prior to study participation, newborns´ mothers signed a written informed consent in local language. As this is a sensitive assessment, we ensured confidentiality and communicated the results of the screening to the parents in an empathetic manner. For the newborn who tested positive, we informed the parents of the presence of SCD. We gave them detailed information about the disease. Finally, we referred them to an appropriate attending physician to provide the necessary care for the child (e.g. penicillin prophylaxis from the age of 3 months and all relevant measures, including confirmation of the diagnosis by a standard method). The research protocol was approved by the “Comité d´Ethique du Nord-Kivu” under the reference: 07/TEN/CENK/2021.

### Study sample

Considering an HbAS prevalence of 23.3% found in Kisangani (the nearest area where NBS was previously performed) [8], the calculated sample was a minimum of 1176 neonates to be tested using the Kish and Leslie formula, with a 95% confidence interval [16]:


$$Sample\text{ size}=\frac{Z_{1-\alpha /2}{}^{2}P\left(1-p\right)}{d^{2}}$$

where Z_1-α/2_=95% confidence interval (standard value of 1.96); p = estimated prevalence of sickle cell disease based on the study by Agasa B *et al*. conducted in Kisangani (DRC) in 2010; d = 5% margin of error (standard value of 0.05).

### Data analysis

We used Epi Info version 7.2.4.0 for data entry and the RStudio version 3.6.3 for data analysis. We used proportions for categorical variables and means for normal variables. To compare the frequencies of studied variables between Hb phenotypes groups, we used the Fisher's exact test. A P-value <0.05 was considered to indicate statistical significance.

## Results

The results of this study show that 1195 newborns were tested (593 females and 602 males) in four major maternity units of the cities of Butembo and Beni: University Clinics of Graben (10.9%), Beni General Referral Hospital (22,6%), Matanda Hospital (28.8%) and Katwa General Referral Hospital (37.7%). The overall results are as follows: 2 newborns (0,2%) were screened positive for hemoglobin SS (1 male and 1 female), 71 (5.9%) for hemoglobin AS (41 males and 30 females) and none positive for hemoglobin C. The anthropometry data showed the following profile: mean birth weight 3031,7 g (min 1740 g, max 4800 g), mean birth height 49 cm (min 43 cm, max 54 cm), mean head circumference 35 cm (min 30 cm, max 52 cm). Eleven neonates (10 HbAA and 1 HbAS) presented the following apparent congenital anomalies: seven with polydactyly, others with cleft lip and palate, clubfoot, macrocephaly and syndactyly. Table 1 shows the socio-demographic characteristics and hemoglobin phenotype of participants. Table 2 shows the distribution of newborns´ hemoglobin phenotype according to mother´s age, ethnicity, marital status, history of SCD testing; newborn´s birth weight, sex and presence or not of apparent congenital anomalies.

**Table 1:** socio-demographic characteristics and hemoglobin phenotype of participants

Characteristic	Number (n=1 195)	Percent (%)
**Mother’s age (years)**		
<18	46	3,8
[18-35]	999	83,6
>35	150	12,6
**Mother’s ethnicity**		
Nande	1147	96
Other*	48	4
**Mother’s history of premarital SCD testing**		
No	565	47,3
Yes	630	52,7
**Mother’s marital status**		
Married	766	64,1
Unmarried	425	35,6
Unknown	4	0,3
**Newborn’s sex**		
Male	602	50,4
Female	593	49,6
**Newborn’s Hb phenotype**		
**HbAA**	1 122	93,9
**HbAS**	71	5,9
**HbSS**	2	0,2

* « Other » includes: Bembe, Budu, Havu, Hema, Hunde, Ikobo, Kumu, Lendu, Lokele, Luba, Mbuba, Mukovo, Mukusu, Mungala, Musoko, Musongora, Muthalinga, Nyanga, Piri, Rega, Shi, Tembo and non specified ethnic group.

**Table 2:** distribution of newborn’s hemoglobin phenotype according to mother’s age, ethnicity, marital status, history of SCD testing; newborn’s birth weight, sex and presence or not of apparent congenital anomalies

	Newborn’s hemoglobin phenotype				P-value
Mother’s age	HbAA	HbAS	HbSS	Total	
<18	39	7	0	46	0,1188
[18-35]	941	56	2	999	
>35	142	8	0	150	
**Total**	**1122**	**71**	**2**	**1195**	
**Mother’s ethnicity**					
Nande	1083	63	1	1147	**0,0015**
Other	39	8	1	48	
**Total**	**1122**	**71**	**2**	**1195**	
**Mother’s marital status**					
Married	727	39	0	766	**0,0304**
Unmarried	391	32	2	425	
Unknown	4		0	4	
**Total**	**1122**	**71**	**2**	**1195**	
Mother’s history of premarital SCD testing*					
No	527	36	2	565	0,2824
Yes	595	35		630	
**Total**	**1122**	**71**	**2**	**1195**	
**Newborn birth weight (gr)**					
< 2500	87	9	0	96	0,345
[2500-4000]	1024	61	2	1087	
>4000	11	1	0	12	
**Total**	**1122**	**71**	**2**	**1195**	
**Newborn’s sex**					
F	562	30	1	593	0,4235
M	560	41	1	602	
**Total**	**1122**	**71**	**2**	**1195**	
**Apparent congenital anomalies**					
No	1112	70	2	1184	0,5016
Yes	10	1	0	11	
**Total**	**1122**	**71**	**2**	**1195**	

* When adjusted to ethnicity, the Nande participants had a significant history of SCD testing (613/1147) compared to others (17/48) with a P-value of 0,01761.

## Discussion

The neonatal prevalence of HbSS (0.2%) and HbAS (5,9%) that we retrieved in this study is lower in this eastern region of the DRC compared to findings in other regions of the country where the following neonatal prevalence was respectively reported for HbSS and HbAS phenotypes: 1.4% and 16.9% in the west and south [7]; 0.96% and 23.3% in the north [8]; 1.9% and 26.8% in the center [17]. We compared these figures with neonatal prevalence in some neighboring countries and we noted a remarkable difference between the east and the west. In the eastern neighboring countries in Rwanda and Burundi, the reported numbers were 0.11% for HbSS and 3.28% for HbAS [9] while in the western neighboring country in Congo Brazzaville, a recent study reported 1.35% for HbSS and 19.43% for HbAS [18].

Regarding the distribution of SCD worldwide and in Africa, several authors have analyzed factors which are associated with it. Among these, malaria endemicity, ethnicity and migration patterns have been largely pointed out [19]. Regarding the lower SCD prevalence in the eastern region of the DRC, the mountain climate which is not favorable to the proliferation of malaria, unlike the other regions of the country, may be the main explanation as North-Kivu displays the lowest malaria prevalence numbers in DRC [20]. However, other internal factors can also dictate the distribution of the disorder within specific areas. For instance, the region of Beni and Butembo where we conducted our study, is home to populations essentially of the Nande ethnic group and we realized that the non-Nande populations were significantly more affected by hemoglobin sickle cell disorders than the Nande participants (P-value=0.0015). Additionally, mothers who reported to be married were less affected (P-value=0.0304). We explained this phenomenon by the fact that the Nande participants had a significant history of premarital SCD testing which may have contributed to reducing the number of risky marriages. Similarly, positive outcomes of SCD premarital screening were noted in Saudi Arabia [21] and Bahrain [22]. However, generalized SCD newborn screening coupled to carriers´/patients´ education remains the gold-standard tool not on

ly to prevent risky marriages but also to initiate an early management program that has proven to improve patients´ quality of life and survival [23].

In this study, we attempted to investigate the possible link between maternal age and the phenotypic manifestation of SCD since many studies have established that extreme parental ages constitute a significant risk factor for the phenotypic manifestation of common hereditary disorders [24-26]. However, no significant association between maternal age and newborn SCD manifestation was noted in this study (P-value=0.1188). We performed a literature search on the effect of parental age on SCD phenotypic manifestation, a single gene disorder, and we found no study that analyzed the association between mother´s age with SCD phenotypic expression in newborns. In order to drive definite conclusions, mother´s hemoglobin status and father´s age must be considered as variables in further studies. We also investigated the possible link between SCD and associated congenital anomalies as birth defects tend to be entangled in most cases. Regarding the association between HbS with the occurrence of other apparent congenital anomalies, there was no significant difference in the different Hb phenotypes (P-value=0.5016). Our findings highlight that SCD may often be an isolated congenital anomaly. However, since many congenital anomalies are not compatible with life (we considered only live and viable babies in our sample) and given we did not find in the literature similar studies to support or refute our findings, further research is thus warranted.

We found no significant difference in hemoglobin phenotype distribution by gender (P-value=0.4235), SCD being an autosomal recessive genetic disorder, hence not gender-related. A similar non-gender-related distribution is ascertained by many NBS studies [17,18]. Regarding the birth anthropometry (especially birth weight) of the newborns in our study, we did not find a significant difference between the three Hb phenotypes (P-value=0.345). As fetal hemoglobin (HbF) has a sparing effect on HbS polymerization which triggers SCD clinical manifestations beyond the neonatal period, homozygous or heterozygous neonates are clinically similar.

**Study limitations:** this is a pilot study that used a tool which was only able to detect three types of hemoglobin: A, S and C. The HbSS or HbAS results were not confirmed by a gold standard diagnostic tool such as high-performance liquid chromatography or isoelectric focusing. However, considering the good field performance of HemopType^TM^, the authors are confident in the results. Due to financial limitation, we were not able to screen parents´ hemoglobin status; thus, we could not analyze its effect on the odds of SCD phenotypic expression in the newborns.

## Conclusion

SCD prevalence was found lower in Butembo and Beni cities compared to other regions of the DRC. However, it remains an alarming public health issue in the context of a lack of policies that regard SCD as a national priority in terms of disease prevention and management. Therefore, we urge decision-makers at national and global level to implement systematic newborn screening, patient/carrier education and early management programs, which should be integrated into the national health system.

### What is known about this topic

- *Newborn screening for SCD is paramount to initiate early management programs that reduce disease severity and improve patients´ quality of life and survival, however, it is not systematically performed in the DRC;*
- *A few studies have already been published on SCD newborn screening in some areas of the DRC (Kinshasa, Lubumbashi, Kindu and Kisangani) and SCD neonatal prevalence varies from 0.96% to 2% for HbSS and 16.9 to 26.8% for sickle cell trait*.

### What this study adds

- *The DRC is a huge country with the highest burden of sickle cell disease in Africa after Nigeria and a paucity of publications, so there is a need for sustained research in various fields pertaining to SCD;*
- *This study provides new data on newborn SCD prevalence in a highland area of the DRC where no data have not previously been published, highlighting a different pattern of SCD prevalence;*
- *The study adds interesting hypotheses on the factors that dictate SCD distribution in this area of the DRC such as the positive effect of premarital SCD screening and ethnical disparity on disease in the community*.
